# Perception of recovery of households affected by 2008 Wenchuan earthquake: A structural equation model

**DOI:** 10.1371/journal.pone.0183631

**Published:** 2017-08-30

**Authors:** Le Lin, Ying Wang, Tianxue Liu

**Affiliations:** 1 Key Laboratory of Environmental Change and Natural Disaster of Ministry of Education, Beijing Normal University, Beijing, P.R.China; 2 Academy of Disaster Reduction and Emergency Management, Beijing Normal University, Beijing, P.R.China; Liverpool School of Tropical Medicine, UNITED KINGDOM

## Abstract

Much of the literature on recovery focuses on the economy, the physical environment and infrastructure at a macro level, which may ignore the personal experiences of affected individuals during recovery. This paper combines internal factors at a micro level and external factors at a macro level to model for understanding perception of recovery (PoR). This study focuses on areas devastated by the 2008 Wenchuan earthquake in China. With respect to three recovery-related aspects (house recovery condition (HRC), family recovery power (FRP) and reconstruction investment (RI)), structural equation modeling (SEM) was applied. It was found that the three aspects (FRP, HRC and RI) effectively explain how earthquake affected households perceive recovery. Internal factors associated with FRP contributed the most to favourable PoR, followed by external factors associated with HRC. Findings identified that for PoR the importance of active recovery within households outweighed an advantageous house recovery condition. At the same time, households trapped in unfavourable external conditions would invest more in housing recovery, which result in wealth accumulation and improved quality of life leading to a high level of PoR. In addition, schooling in households showed a negative effect on improving PoR. This research contributes to current debates around post-disaster permanent housing policy. It is implied that a one-size-fits-all policy in disaster recovery may not be effective and more specific assistance should be provided to those people in need.

## Introduction

As a severe regional environment event, an earthquake causes dramatic changes in the natural environment and human society. Facilitating recovery, in order to regain sustainability in daily life and in economic activity, after a disaster is an important priority for affected families, the local community and the wider society. The Sendai Framework for Disaster Risk Reduction issued by the United Nations called for a broader and more people-centered approach to reduce disaster risk [1]. This framework calls for four priorities for action, i.e. understanding disaster risk, strengthening disaster risk governance to manage disaster risk, investing in disaster risk reduction for resilience, enhancing disaster preparedness for effective response and to “Build Back Better” in recovery, rehabilitation and reconstruction. The import roles of stakeholders, international cooperation and global partnerships are also addressed in this framework. Each individual endures and perceives the impact of a disaster differently, perception of recovery is judged subjectively based upon personal experience. Whether recovery is effective is not only viewed or evaluated by the government; it is also the subjective judgment of affected individuals who sense different levels of impact and losses from an extreme event [2]. According to Quarantelli [3], the core of recovery may vary in different research backgrounds. The concept of recovery implies attempting to bring the post-disaster situation to some level of acceptability. The notion of recovery may cover four terms started with the letter R, i.e. reconstruction (exclusively on the rebuilding of the physical infrastructures destroyed or damaged in a disaster), restoration (re-establishing physical and social patterns), rehabilitation (rebuilding to a higher standard than before the disaster) and restitution (legal actions to return to a former state of affairs). In all societies, the family is the basic unit of social organization [4], which refers to a statement about re-establishing pre-impact physical and social patterns [3]. For the purpose of assessing the perception of recovery (PoR), the concept of recovery in this study is regarded as an outcome rather than a process.

Previous research suggests that governments and citizens have different perceptions of recovery [5–7]. Long-term recovery studies on the Kobe earthquake [6, 8] revealed that a substantial discrepancy existed between government and citizen perceptions of recovery: the city government perceived the city to be completely recovered ten years after the earthquake, whereas the citizens perceived the city to be only 80% recovered.

Recent research has gradually begun to examine the perception of recovery and adaption following natural disasters [2, 5, 9]. Research on PoR can occasionally overlap with research on the recovery process because the two phenomena share explanatory factors. However, the research on PoR may focus more closely on the subjective assessment of recovery quality by disaster victims, which can reflect the formation of life satisfaction after dramatic regional social change against a given socio-economic background. In contrast, the research on recovery as a process may pay more attention to the objective assessment of recovery efficiency and effectiveness, which can reflect the multiple requirements of the so-called “good recovery”.

In extant studies about recovery, many were conducted at a macro level to determine persuasive statistical indicators that could be used to calculate the degree of recovery, such as urban disaster recovery from earthquake [10], housing recovery after a hurricane [11] and inequity in recovery [12]. However, this paper intended to determine how to evaluate perception of recovery of those affected households and what factors have significant influence on such perceived satisfaction. Studies of perception of recovery for household mainly focused on the measurement of PoR [9, 13] and the influencing factors of PoR [13, 14]. A sociological study by Fothergill [15] provided a vivid picture of how women played a role in the recovery of their families following the 1997 floods in North Dakota and Minnesota, in the U.S.A. Fothergill showed the importance of household activities and attributes (such as accepting charity, recreating domestic culture, and social roles) in the recovery process, which implied the necessity of taking internal factors associated with perceived recovery into consideration. While investigating the recovery following a flash flood that occurred in Buffalo Creek (West Virginia, U.S.), Erikson [16] demonstrated that the replacement of communities with trailer parks in the post-disaster period had made recovery for survivors more difficult, because many were unable to reestablish close personal relationships. In his work, the impact caused by external players such as the government was emphasized.

From these previous studies, conditioning factors on perception of recovery can be grouped into internal factors at the micro level (i.e. those subject to the will of households) and external factors at the macro level (i.e. those not subject to the will of households). Starting from this basis, this paper tried to combine internal factors at the micro level and external factors at the macro level to construct and test a model for the perception of recovery. This research also aimed at discovering conditioning factors of PoR and interactions of these conditioning factors in the PoR model.

## Research background

### The 2008 Wenchuan earthquake

On May 12, 2008, a large earthquake of magnitude 8 on the Richter scale occurred in the Wenchuan area of Sichuan Province, China. It was the worst earthquake to occur in China since the M7.8 Tangshan Earthquake in 1976. The earthquake also caused severe secondary disasters, such as landslides, rock falls and debris flows [17–19]. By August 25, 2008, the Wenchuan earthquake had caused 69,226 deaths and 374,643 injuries. In addition, 17,923 people were missing [20].

**Fig 1:**
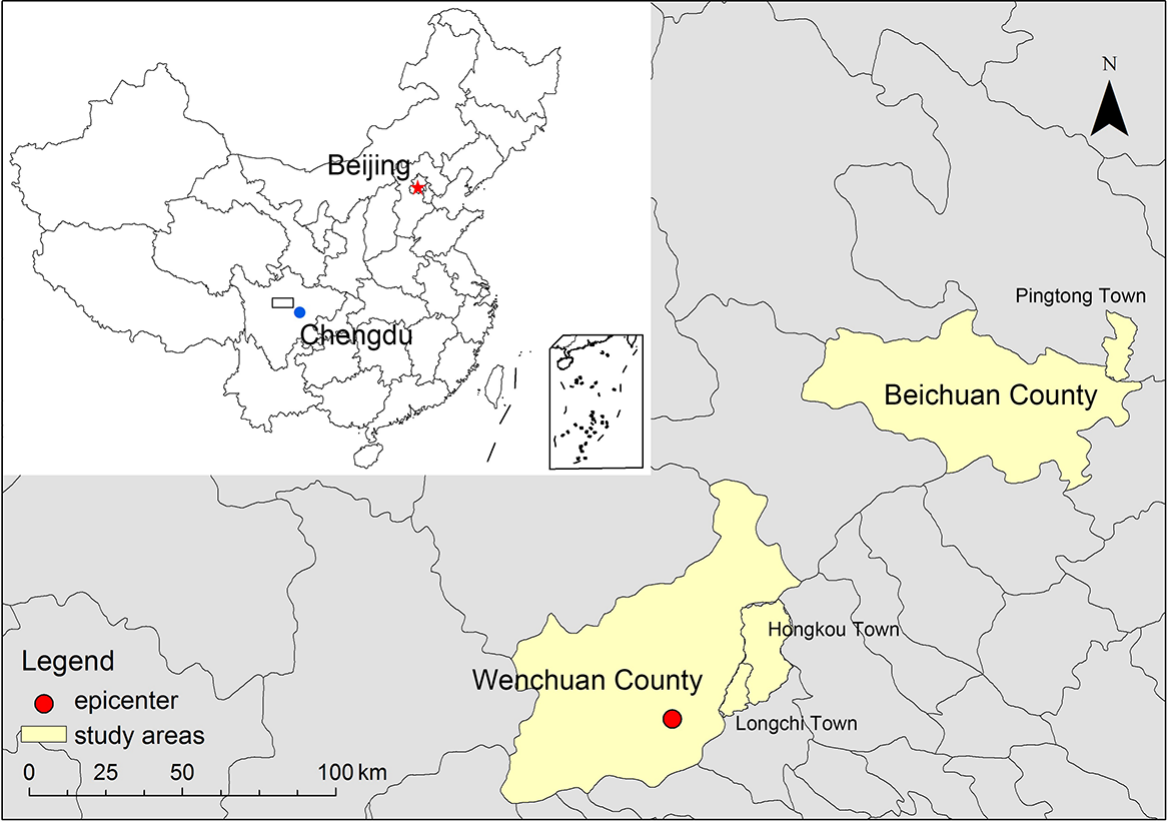
Location of study areas.

In the post-earthquake loss assessment, Wenchuan County and Beichuan County (as shown in Fig 1) were included in the top 10 disaster-hit counties. These two rural areas were under developed but suffered severe loss in this earthquake, both physically and socially (as shown in Table 1, data of disaster loss were extracted from [21–22]). The 2008 Wenchuan earthquake severely damaged a large number of residential buildings in these two areas, which made it challenging for the affected households to regain housing and re-establish new lives. Hence they were ideal samples for research on household recovery from an earthquake.

**Table 1:** Background of two rural areas damaged in the 2008 earthquake.

Area	Households in 2007 (10000 people)	Rural population ratio in 2007	GDP per capita in 2007 (in RMB)	Death and missing	Collapsed house (per 10000 people)	Sample amount (households)	Response rate
Wenchuan County and surrounding towns	27 523	63.8%	26 204	23 871	55 291	345	78.59%
Beichuan County and surrounding towns	41 211	86.9%	8 598	20 047	21 741	266	60.59%

### Variable selection

As the dependent variable in the model, the perception of recovery (PoR) was defined as the subjective assessment of the degree of recovery by the disaster affected family. In the existing literature, subjective assessment by interviewees of household recovery has been commonly used because it reflects the core of PoR in self-evaluation and subjectivity [9;23–25]. In these assessments, the sense of satisfaction is quite difficult to be accurately described by numerical data. This was different from other assessments using quantitative statistical indicators to describe the recovery process. In the consideration of recovery, quality and time were two metrics most frequently mentioned for the evaluation and success of recovery interventions [26, 9]. Hence self-evaluated quality of life (SEQoL) and self-evaluated recovery speed (SERS) were included as observed variables of PoR. In this research, SEQoL means the general quality of life of the household if compared with that before the earthquake, and SERS means the recovery speed that family members feel occurred compared with that for other surrounding families. Furthermore, much of the literature has stressed psychological or psychosocial recovery [27–29]. In Fothergill’s research [15], the importance of recreating domestic culture was mentioned. Hence family atmosphere (FA), which focused on the mental health of family members and family bonds was included in the building of PoR. In this paper, FA means the general atmosphere in affected households. Thus, for the latent variable PoR, three observed variables were selected, self-evaluated quality of life (SEQoL), self-evaluated recovery speed (SERS) and family atmosphere (FA).

As one independent latent variable in the model, house recovery conditions (HRC) was defined as the measurement of external conditions and difficulties each affected family faced to achieve housing recovery. The retaining of dwellings plays a significant role in the process of disaster recovery. From the Northridge and Kobe earthquakes, it was learned that housing losses could have devastating effects on the recovery effort [4]. On the one hand, the extent to which a house is damaged in the earthquake can define the difficulties of housing recovery [30]. Hence house damage degree (HDD) was included to represent the damage caused by the earthquake. In this study, HDD means the condition of affected dwellings right after the earthquake. On the other hand, housing and shelter policies can have a significant impact on recovery [31]. In the research of Rathfon [32], building damage and reconstruction policy were both included in the assessment of housing recovery. In response to the top-down recovery plan designed by the central government in China [33], the local governments in disaster-hit regions deliver highly detailed recovery and reconstruction plans for their regions. Within these local recovery plans, households are required to rehouse in different ways, such as independently raising funds and rebuilding, or settling in dwellings provided by the government. Different types of reconstruction policies may introduce different difficulties for affected families and produce different economic cost. Hence rebuilding method (RM) was included. In this research, RM means the paths or policies that the affected households apply for regaining permanent dwellings. Thus, for the latent variable HRC, house damage degree (HDD) and rebuilding method (RM) were selected as the observed variables.

As one independent latent variable in the model, family recovery power (FRP) was defined as the measurement of internal conditions and drivers each affected family had to achieve a good recovery. Family recovery power (FRP) can be a socio-economic attribute that is closely related to household recovery and plays an important role in the recovery process [30, 34]. FRP possibly includes public funding, household income, human resources for labour work and educational attainment as conditioning factors. It was found that in the research on natural disasters, family attributes such as labour, income and education were regarded as components of family resilience or coping capacity against disaster [35–37]. Accordingly, family attributes in this study were collectively named as family recovery power with the background of disaster research. Low-income individuals and families were the most severely disadvantaged and marginalized groups during the first three years of recovery that followed the Great Hanshin Earthquake [35]. Longitudinal studies of family recovery from a flash flood in the U. S. and an earthquake in Nicaragua revealed that the perception of recovery in the U.S. disaster could be best explained by losses, aid received and recovery of pre-disaster income levels [14]. Hence household’s income change (HIC) was included as an observed variable for FRP. In this paper, HIC means the change of net income of the affected households compared with the net income before the earthquake. In research on a resilient community after Hurricane Katrina, labour resources could significantly affect recovery quality [36]. In addition, it was discovered that unemployment is a significant influencing factor with respect to disadvantaged households in disaster recovery [37]. Hence post-earthquake labour-work population (PeLWP) was included. In this study, PeLWP means the number of household members of the affected households who went to work as labourers (such as those in infrastructure industries) after the earthquake. Schooling condition is an important component in social capital [38–39]. Social capital could operate across several different fields of practice such as family, schooling, work, and recreational activities [40–41]. Hence, education condition (EC) which mainly investigated schooling in affected families was included. In this research, EC means the general assessment of the household members who are being educated in schools. Thus, household’s income change (HIC), post-earthquake labour-work population (PeLWP) and education condition (EC) were selected as the observed variables for the latent variable FRP.

In addition, it was discovered that there were some special factors that could be influenced by both external and internal factors during recovery. In this research, reconstruction investment (RI) was defined to play such a role, and it represented the actual monetary input affected families spent to finish recovery. Apparently, RI may be closely related to HRC. A better HRC reduces rebuilding difficulties and reconstruction input. However, RI can also be closely related to FRP. The monetary value of investment made in a housing unit over the recovery period can be significantly influenced by factors such as permanent economic income, current economic resources and dwelling condition [42]. Therefore, RI may bridge the gap between FRP and HRC. It should be included in the PoR model to be comprehensive.

Generally, PoR was set to represent the main target of this research, the perceived recovery of households after a disaster. HRC, FRP and RI served as explanatory variables to PoR, while HRC and FRP addressed the external and internal factors of households, which may influence how PoR was formed. RI can be special but necessary for its influence on PoR and its connecting role between HRC and FRP.

## Methods

### Ethics statement

Prior to conducting our study, we received approval from the Human Subjects Committee of the School of Social Development and Public Policy (SSDPP-HSC), Beijing Normal University (BNU). Written informed consent was obtained from each of the participants prior to being interviewed. Each of the participants was free to withdraw from this survey at any time without any consequence.

### Field survey

This study sought to address the research gap in the PoR field by examining how HRC, FRP and RI influence a household’s perception of recovery using the questionnaire survey method. In the designed questionnaire, most items were ranked on a Likert scale [43], and the related responses were converted into ordinal variables. Only a few items were designed as continuous variables, such as post-earthquake labour-work population (PeLWP). More details about the survey questionnaire can be found in the supplementary materials.

There were two investigations in this research. The first one, pre-survey, was conducted in August 2012, approximately 4 years after the earthquake. The second one, the main investigation lasted approximately two weeks and was conducted in January 2014, approximately 5 years after the occurrence of the earthquake. Samples in this research came from the second survey, with a total of 878 households. Eleven experienced investigators who had participated in other disaster surveys and were well trained before departure participated. The geographic extent of this investigation covered the densely populated towns of two rural areas stricken severely by the earthquake (as shown in Fig 1).

When deciding the proper sample size, all the disaster-affected households in the two rural areas were taken as the population for the survey. To meet the standard of a 95% confidence level and a 5% sampling error (i.e., 4 weeks) [44], the proper sample size of each area is at least 200 households. Based on random sampling in the main towns of those two areas, surveys were conducted face-to-face in local households and with the head of each household (if the head was not accessible, then a family member who knew the recovery process was asked to answer). Finally, a total of 611 well-documented sample households were collected.

### Structural equation modeling

Structural equation modeling (SEM) is a widely used multi-variable statistical method in the field of behavior research and social science. It enables the researcher to explicitly model measurement errors and may therefore result in less biased parameter estimations, which is an advantage over multiple regression and path analysis. Although techniques such as principal component analysis (PCA) can decrease the dimensionality of a set of correlated variables using a smaller number of axes (i.e., latent variables), PCA cannot be performed *a priori* on the basis of theoretical rather than empirical considerations.

The basic assumption when using SEM is the same as when using other multi-variable methods: the population is required to follow the hypothesis of multivariate normality [45]. On this basis, the most popular estimation method is maximum likelihood (ML), followed by general least square (GLS) [46]. When the sample data does not follow the hypothesis of normality, estimation using GLS can function to analyze small sample data [47]. Because the sample data in this research are primarily categorical and the sample amount is under 1 000, this paper uses GLS to perform model estimation.

SEM is a confirmative analysis method, which should be supported and guided by related theory and a developed *a priori* model (displayed in the following section on model specification). During the construction of the structural equation model, a series of technical tasks were performed. First, a descriptive statistics analysis of each variable was done and a table of correlation coefficients was generated (Table 2) to investigate the relationship among those observed variables to have better model specification. Then, to additionally investigate the relationship among the variables, SEM with GLS estimation was implemented using SPSS AMOS 22 (IBM, Armonk, NY). To examine the performance of outcome models, several indices of model fit were assessed: the chi-square test, absolute index for goodness of fit, the added index for goodness of fit and the simple index for goodness of fit. A comprehensive list of testing indices and related critical values [48–50] are provided in Table 3. Because the data is collected in ordinal form, Kendall rank correlation coefficients [51] are thereby presented in Table 2.

**Table 2:** Correlations between observed variables (n = 611).

	EC	HIC	PeLWP	HDD	RM	SEQoL	SERS	FA
**HIC**	-.084*							
**PeLWP**	-.084*	.167**						
**HDD**	-.032	-.009	-.032					
**RM**	-.007	-.034	-.055	.337**				
**SEQoL**	-.082*	.260**	.090**	.061	.042			
**SERS**	-.045	.087*	.068	.151**	.082*	.188**		
**FA**	-.023	.099**	.025	.001	.008	.218**	.212**	
**RI**	-.044	.032	.138**	-.236**	-.208**	.034	.061	.023

*p < .05 (2-tailed)

**p < .01 (2-tailed)

**Table 3:** Test results of model built and comprehensive critical values.

Statistical testing index	Standard for adaption	Test results	Adapted model^a^
*χ*^2^/*df*	<3	1.681	Y
P value	>.05	.024	N
Absolute index for goodness of fit			
RMR	< .05	.244	N
RMSEA	< .08	.033	Y
GFI	>.90	.987	Y
AGFI	>.90	.972	Y
Added index for goodness of fit			
NFI	>.90	.859	N
IFI	>.90	.938	Y
TLI(NNFI)	>.90	.892	N
CFI	>.90	.934	Y
Simple index for goodness of fit			
PGFI	>.50	.482	N
PNFI	>.50	.525	Y
PCFI	>.50	.571	Y

^a^ Y for passing the related test and N for not passing

### Model specification and basic hypothesis

**Fig 2:**
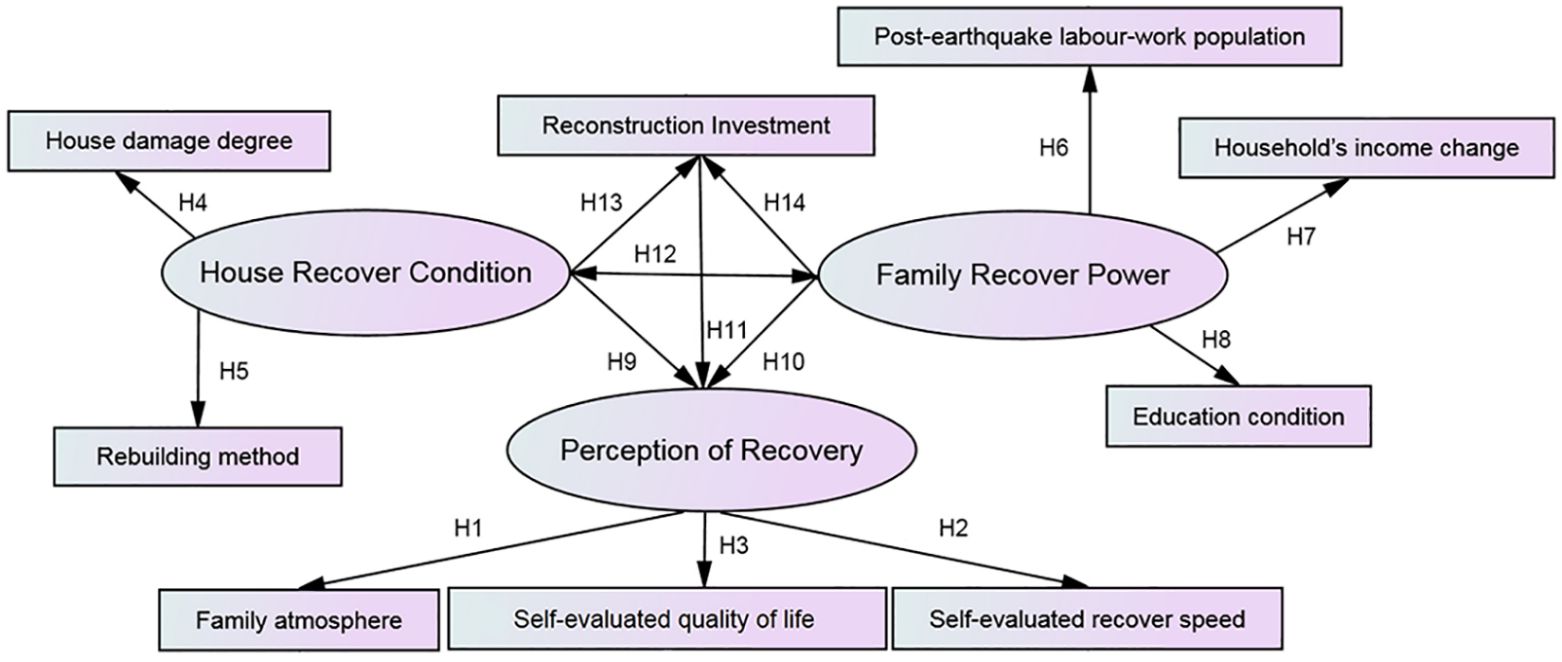
Model specification. Solid arrows represent hypothesized paths.

When investigating with SEM, latent variables (which cannot be measured by questionnaire) and observed variables (which can be measured in a survey) are required to construct a hypothesis model (Fig 2). When constructing the model, a number of hypotheses were made. For FA, it was assumed that the more that the affected family could eliminate an atmosphere of desperation and face life optimistically, the higher the level of PoR there can be (Hypothesis 1, H1 for short). For SERS, it was assumed that the faster that the affected families recovered, the higher the level of PoR they may have (H2). For SEQoL, it was assumed that if the affected families agreed that their post-earthquake quality of life was better than their pre-earthquake quality of life, their PoR could be higher (H3).

For HDD, it was assumed that the worse HDD that a family experienced, the lower the HRC it may have (H4). From the items in HDD, it was discovered that these items were about how bad the damage was in the house and thus could be negative to PoR but to a different extent. For instance, houses without any need for repaire could easily help the affected families recover to pre-disaster life, while completely damaged house could be heavy burdens on the affected families, for they had to earn more to rebuild. Because of the negative effect of house damage on PoR, HDD was coded from -4 to -1, illustrating the damage degree from severe to slight. For RM, a complex and costly reconstruction method could be negative for recovery perception. It was assumed that the fewer burdens that a family carried during rebuilding, the higher HRC they may have (H5). For instance, simply repairing may cost the affected house in monetary input, while the policy of planning by the government and building alone could make the affected households complete the series of administrative procedures and thus cause mental and physical fatigue. Because of the negative effect of the rebuilding method on PoR, RM was coded from -5 to -1, illustrating the reconstruction method from complicated and costly to simple and inexpensive.

For PeLWP, it was assumed that the more members of the affected families there were who could work and earn money, the higher FRP they might have (H6). For HIC, it was assumed that if the total income of a family could be more than before the disaster, the higher the level of FRP it could have (H7). EC was an important component of social capital [38–39]. It was assumed that more schooling could lead to better social capital, leading to higher the level of FRP (H8).

In addition, several hypotheses were proposed regarding the contributions of HRC, FRP and RI to PoR and the interactions between HRC, FRP and RI. From the correlation analysis of the observed variables, it was discovered that HDD and RM (components of HRC) had a positive correlation with SEQoL, SERS and FA (components of PoR) as well as HIC, PeLWP and EC (components of FRP). It may mean that good HRC and strong FRP could improve the level of PoR. Additionally, a high level of RI demonstrated that the affected family had been equipping a new house and certainly would result in better living conditions, which could improve PoR. Therefore, it was assumed that HRC, FRP and RI could have a positive direct connection with PoR (H9, H10, H11), whereas a positive correlation was assumed to exist between HRC and FRP, between HRC and RI and between FRP and RI (H12, H13, H14).

## Results

### Description

First, a descriptive statistical analysis on observed variables was performed to obtain a general view of the sample families (Table 4). The analysis revealed that more than half of the investigated families were experiencing a good atmosphere at home and had recovered from the earthquake impact, psychologically. Approximately 50% of the surveyed families thought that their recovery speed was the same as that of others, and approximately 80% thought that their living conditions were not worse than before the disaster. Regarding RI, most families invested no more than 300 thousand RMB into regaining permanent houses, with 80 thousand to 160 thousand RMB being the most frequently expended sum. Regarding FRP, nearly 95% of the surveyed families had at least one member labouring for money, and 50% had at least two. Of the surveyed households, 50% had more economic income than before the earthquake, whereas 30% had less income. Over 60% of the households had no family members studying in schools higher than primary school when the survey was administered. It was uncommon to see households with many children studying in high schools or universities. Regarding HRC, more than 80% of the surveyed households claimed that their houses were severely damaged or had collapsed, whereas approximately 90% of the sample households intended to rebuild their houses. Among those who had determined to rebuild, different rebuilding methods were adopted: 45% were rebuilding independently or buying commercial apartments, 25% were independently building government-planned houses and 20% were resettled fully by the government.

**Table 4:** Descriptive statistics of observed variables in sample households (n = 611).

Variable	Value	Count	Ratio (%)	Variable	Value	Count	Ratio (%)
EC	0	381	62.36	PeLWP	0	48	7.86
	1	63	10.31		1	248	40.59
	2	64	10.47		2	211	34.53
	3	70	11.46		3	77	12.6
	4	14	2.29		4	23	3.76
	5	10	1.64		5	3	0.49
	6	9	1.47		6	1	0.17
HIC	1	180	29.46	HDD	-4	495	81.02
	2	122	19.97		-3	92	15.06
	3	309	50.57		-2	12	1.96
					-1	12	1.96
RM	-5	275	45.01	SEQoL	1	41	6.71
	-4	152	24.88		2	88	14.4
	-3	124	20.29		3	202	33.06
	-2	47	7.69		4	235	38.46
	-1	13	2.13		5	45	7.37
SERS	1	68	11.13	FA	1	29	4.75
	2	152	24.88		2	56	9.17
	3	293	47.95		3	189	30.93
	4	82	13.42		4	322	52.7
	5	16	2.62		5	15	2.45
RI	[0,80)	140	22.91				
	[80,160)	262	42.88				
	[160,300)	158	25.86				
	[300,1000)	51	8.35				

### Structural equation modeling

**Fig 3:**
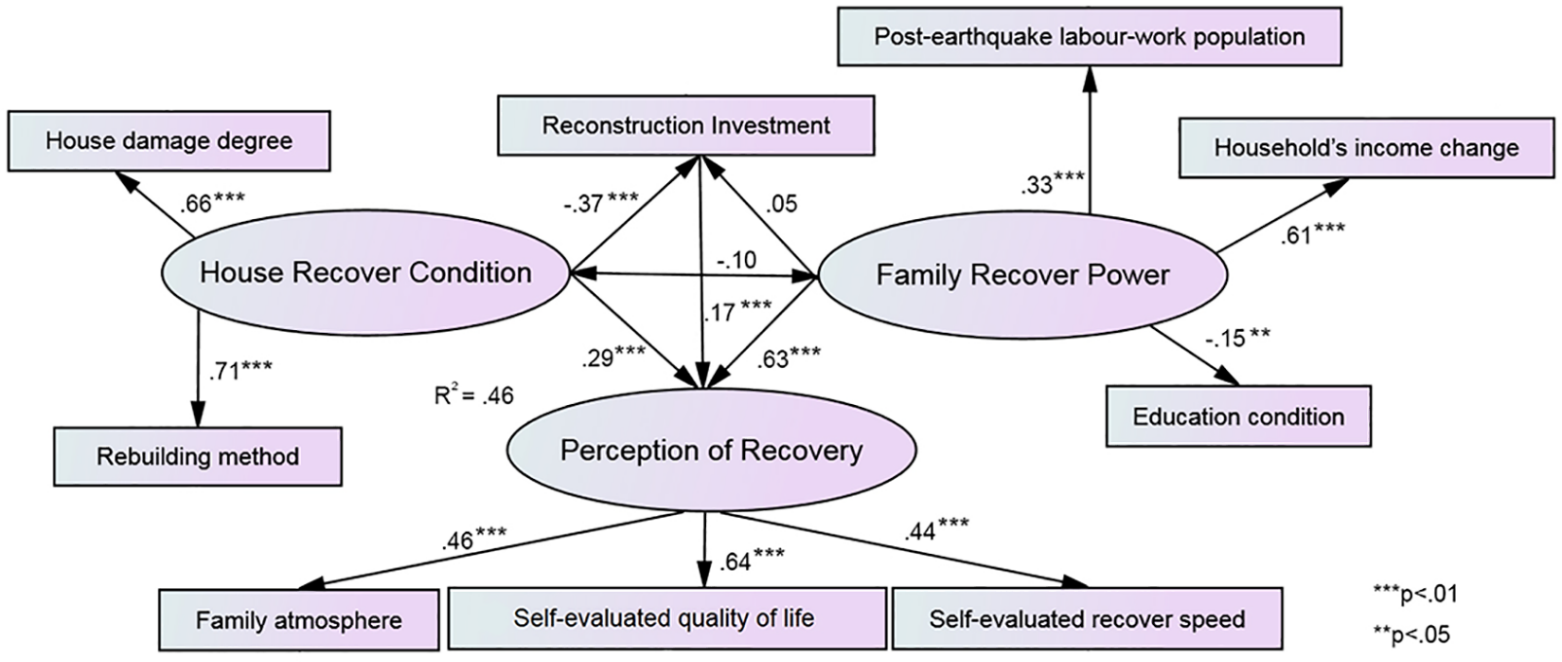
SEM model of perception of recovery.

The process of model-building and estimating was completed using the statistical software SPSS AMOS 22 (IBM, Armonk, NY), and the model result can be found in Fig 3. The tests of goodness of fit shown in Table 3 illustrated that the hypothesis model in this research was supported by the investigated sample data. Thus, this model was acceptable.

The path weights of the observed variables for the latent variables are as shown in Fig 3. Standardized as opposed to unstandardized path coefficients are reported here because the relative importance of different paths was of primary interest [52]. Regarding PoR, the most influential item in its formation was SEQoL, with a regression weight of .64, followed by FA (.46) and SERS (.44). Regarding HRC, both RM (.71) and HDD (.66) strongly promoted this latent variable. For FRP, HIC weighed the most (.61), followed by PeLWP (.33). Surprisingly, the education condition negatively influenced FRP (-.15). Therefore, with the model established, most hypotheses (H1-H7) were supported, with the exception of H8, which implied that more schooling with the household could harm FRP.

Regarding the contributions and interactions of the three conditioning aspects, it was found that hypotheses H9, H10 and H11 were supported. FRP, HRC and RI contribute significantly to PoR, with regression weights of .63, .29 and .17, respectively. Generally, the model constructed using SEM, which was intended to explain PoR with respect to the three aspects RI, HRC and FRP, was reasonable. The model could explain 46% of the total variance in the dependent variable. Unexpectedly, the relationships between HRC and FRP and between FRP and RI were not significant, which implied that hypotheses H12 and H14 were not supported. In addition, HRC had a significant negative influence on RI (-0.37), which implied that hypothesis H13 was not supported.

Based on the model result, HRC as the direct sequential factor from the earthquake had substantial influence on PoR but was outweighed by FRP when considering its effect on PoR. However, the education condition failed to play a positive role in strengthening FRP for better PoR. There were significant interactions between HRC, RI and PoR. HRC had a negative direct influence on RI. RI had a positive direct influence on PoR. However, HRC had a positive connection with PoR. The role of HRC may appear confusing. Based on the model result, there was no significant relationship between FRP and HRC or between FRP and RI.

## Discussion

The results of SEM modeling imply that a positive response by the affected families to challenges in recovery truly matters. Actions such as having a large workforce and seeking to generate more economic income can be effective methods to improve FRP. For limited public resources, more investments in boosting the household’s capacity could lead to relatively higher PoR. As recommended by the World Bank, the housing owner-driven reconstruction (ODR) approach, which could be summarised as giving conditional financial assistance, accompanied by regulations and technical support aimed at ensuring that houses are built back better, is regarded as the best strategy for post-disaster housing provision [26, 53]. Such methods are also supported by the Sendai Framework of Disaster Risk Reduction. The recovery, rehabilitation and reconstruction phase is a critical opportunity to “Build Back Better”. In order to make communities resilient to disaster, strategies for disaster risk reduction including the principles associated with "Building Back Better" need to be integrated into guidelines related to contemporary land use planning and construction standards. The results of this research may reflect these recommendations above. A good recovery policy must provide incentives for affected families to positively engage in the recovery process.

Economically, the return period on education investment is so long that no significant gain is available short term (in a typical family, 20–30 years can be required to produce a university graduate). This phenomenon is so wide spread that most households that survived the earthquake have many consuming members but few productive members. If a family member was of the proper age for employment but still studying at a university, the family would have to feed one more member with one fewer member earning. In such circumstances, a higher schooling population means a heavier economic burden, which could be why the education condition played a negative role in PoR.

The dual effect of HRC was also reflected in this model. On the one hand, a household trapped in worse HRC could invest more money in recovery. The dwellings they had and the facilities at home consequently improved and these conditions could raise their mental wellbeing after such arduous work. Then the affected families could perceive the effort they exerted to improve their lives. On the other hand, if one household had to face worse conditions of post-earthquake housing, they might have to face more challenges than do their neighbors. These difficulties could result in a low level of PoR. From the model results, of the two effects, the latter is predominant.

### Limitations

As mentioned above, the SEM model in this study could explain 46% of the total variance in the dependent variable. To explain the additional 54% of the variance could be the next focus of this research. Improvements could be conducted by enriching the observed variables of the related latent variables. First, PoR is a widely relevant topic with multiple aspects. How to select comprehensive and proper indices should be further discussed. This paper uses FA, SERS and SEQoL as three compound elements of PoR. Other factors, such as living convenience [14], sense of satisfaction and sense of community [54] could also be included. To clarify residents’ perspectives about long-term recovery after Hurricane Katrina, Schumann [13] discovered that memory and mobility guided the formation of residents’ recovery meanings and assessments, which was instructive to improve the measurement of PoR. Second, HRC may also possess the additional aspect of disaster-victim relocation [9] because relocation means more than just adjusting to a new social and natural environment. It also refers to the inconvenience of living distant from employment, schools, and social networks [55]. Third, FRP could also include content, such as funding from relatives and surrounding communities [56]. Whereas kinship and community aid are important in determining the speed and extent of familial recovery, the extent of the contributions from such sources has not been systematically assessed [30]. Schumann [13] discovered that place attachment, life stage, and migration experience factored heavily into residents’ recovery perspectives and these factors could be considered in FRP in further research. In addition, the recovery stage could be helpful [57]. Furthermore, social capital is an inevitable aspect of FRP. The importance of social capital during disaster recovery has been discussed [58–60]. Finally, regarding RI, its components can be enriched, e.g., by including infrastructure investment and decoration input, to obtain a wider view of how the affected families spend their money.

## Conclusion

Using field survey data from two rural areas (Wenchuan, Beichuan) in regions disastrously afflicted by the 2008 Wenchuan earthquake in China, this paper focused on assessing the perception of recovery (PoR) in the affected families. Because of the substantial importance of housing recovery in the entire recovery process, this paper departed from the perspective of rehousing and chose three aspects, house recovery condition, family recovery power and reconstruction investment, to build a household PoR model by applying structural equation modeling. It was discovered that all these three aspects could effectively explain the formation of the affected family PoR. Among the three aspects, family recovery power made the largest contribution, followed by house recovery condition. A significant correlation between house recovery condition and reconstruction investment was found, which reflects the dual effect of house recovery condition. In addition, family recovery power did not significantly influence reconstruction investment.

Findings from this study basically agree with some conclusions in the extant research about the relationship among perception, household attributes and damage severity. In the research on Hurricane Katrina from Khunwishit and McEntire [2], it was found that social vulnerability, which may include class, occupation, employment, poverty, ethnicity, gender, disability, health status, and age, has a statistically significant, positive effect on perceived disaster impact. In addition, they concluded that those who live in areas that are severely damaged by a disaster tend to experience a greater impact than those whose living areas are less damaged or not damaged. In this research, we proved that family recovery power, which comprises labour sources and household income, can improve the perception of recovery and house damage condition can degrade this perception.

As for the method of housing reconstruction, this study proved that different reconstruction methods can have different effects on a household’s perception of recovery. The variable of rebuilding method was divided into five categories and was proven to be significantly influential. Such a finding reflects the research conclusion by Andrew [9], which compared the perceptions of households provided with either in situ housing assistance or resettlement/relocation housing assistance and found that the beneficiaries of in situ housing assistance programs fared better than beneficiaries of the resettlement programs despite the former receiving lower monetary assistance. This finding may lead to further discussions on how to provide good housing policies in the post-disaster reconstruction period.

As an important aspect of a comprehensive assessment of disaster recovery quality [55], a better understanding of PoR can broaden the view of developing post-disaster public services. It is suggested that a one-size-fits-all approach to individual and community disaster recovery, although perhaps expedient, is unlikely to achieve the comprehensive sustainable recovery that is the hope of such programs. As recommended by the World Bank [53], housing reconstruction is key to disaster recovery, but it depends on the recovery of markets, livelihoods, institutions, and the environment. Diverse groups need diverse solutions. Owners are almost always the best managers of their own housing reconstruction. When the majority of affected homeowners owned the land and intended to rebuild on the same sites, owner-driven housing reconstruction seemed the best choice for the challenge [60]. To succeed, policymakers must choose from a wide array of possibilities and tailor their programs to suit the local conditions and socio-political will [9].

## Supporting information

S1 File Data collected from field survey. (XLSX)

S1 Table Survey variables in questionnaire and the classification of responses. (DOCX)
