# Supplementary material for: Perception of recovery of households affected by 2008 Wenchuan earthquake: A structural equation model
Source: PLoS One. 2017 Aug 30;12(8):e0183631. doi: 10.1371/journal.pone.0183631 (PMC5576748; doi:10.1371/journal.pone.0183631)
Supplement: S1 Table — (DOCX) [file pone.0183631.s002.docx]

**S1 Table. Survey variables in questionnaire and the classification of responses.**

| Variable | Question asked in questionnaire | Response options (value assigned) |
| --- | --- | --- |
| Perception of Recovery (PoR) | | |
| Family atmosphere (FA) | How is the atmosphere in your family now? | not recovered yet, still in sadness (1)  not recovered yet, but well (2)  recovered basically, sometimes in sadness (3)  recovered totally, has recovered from the earthquake impact, psychologically (4) |
| Self-evaluated quality of life (SEQoL) | How is the general quality of life of your family now, compared with that before earthquake? | much worse (1)  slightly worse (2)  basically the same, not changed (3)  slightly better (4)  much better (5) |
| Self-evaluated recover speed (SERS) | How do your family members feel about your recovery speed, compared with other surrounding family? | much slower (1)  slightly slower (2)  basically the same (3)  slightly faster (4)  much faster (5) |
| House Recover Condition (HRC) | | |
| House damage degree (HDD) | What is the condition of your dwelling after the earthquake? | generally not affected by earthquake (-1)  slightly damaged, no need for repairing(-2)  severely damaged, need repairing (-3)  completely destroyed, need rebuilding (-4) |
| Rebuilding method (RM) | In what way you regain permanent dwelling? | no amendment(-1)  reinforcement and repairing(-2)  resettled by government(-3)  planned by government and built by self(-4)  built by self or buying commercial apartment(-5) |
| Family Recover Power (FRP) | | |
| Post-earthquake labour-work population (PeLWP) | How many family members go to do labour works after the earthquake? | *this variable is numeric* |
| Household’s income change (HIC) | How has your family net income changed when compared with before earthquake? | decreased (1)  same (2)  increased (3) |
| Education condition (EC) | Investigator may get to know the EC in such family and assign scores to different education level for every schooling member and then sum up scores | *this variable is ordinal*  scoring rules:  if a member is studying in  junior high school or equivalent (1)  senior high school or equivalent (2)  university or equivalent (3) |
| Reconstruction Investment (RI) | | |
| To regain your permanent dwelling, how much money (in thousand RMB) have you paid for repairing or rebuilding? | | *this variable is numeric* |
